# Phenotypic Characterization of Larval Zebrafish (*Danio rerio*) with Partial Knockdown of the *cacna1a* Gene

**DOI:** 10.1007/s12035-019-01860-x

**Published:** 2019-12-26

**Authors:** Kinga Gawel, Waldemar A. Turski, Wietske van der Ent, Benan J. Mathai, Karolina J. Kirstein-Smardzewska, Anne Simonsen, Camila V. Esguerra

**Affiliations:** 1 Chemical Neuroscience Group, Centre for Molecular Medicine Norway, Faculty of Medicine, University of Oslo, Gaustadalléen 21, Forskningsparken, 0349 Oslo, Norway; 2 Department of Experimental and Clinical Pharmacology, Medical University of Lublin, Jaczewskiego St. 8b, 20-090 Lublin, Poland; 3 Faculty of Medicine, Institute of Basic Medical Sciences and Centre for Cancer Cell Reprogramming, Institute of Clinical Medicine, University of Oslo, 1112 Blindern, 0317 Oslo, Norway; 4 School of Pharmacy, Faculty of Mathematics and Natural Sciences, University of Oslo, Sem Sælandsvei 24, 0371 Oslo, Norway

**Keywords:** Zebrafish, *CACNA1A* gene, Loss of function, Epilepsy, Touch response, Antiseizure drugs

## Abstract

The *CACNA1A* gene encodes the pore-forming α1 subunit of voltage-gated P/Q type Ca^2+^ channels (Ca_v_2.1). Mutations in this gene, among others, have been described in patients and rodents suffering from absence seizures and episodic ataxia type 2 with/without concomitant seizures. In this study, we aimed for the first time to assess phenotypic and behavioral alterations in larval zebrafish with partial *cacna1aa* knockdown, placing special emphasis on changes in epileptiform-like electrographic discharges in larval brains. Whole-mount in situ hybridization analysis revealed expression of *cacna1aa* in the optic tectum and medulla oblongata of larval zebrafish at 4 and 5 days post-fertilization. Next, microinjection of two antisense morpholino oligomers (individually or in combination) targeting all splice variants of *cacna1aa* into fertilized zebrafish eggs resulted in dose-dependent mortality and decreased or absent touch response. Over 90% knockdown of *cacna1aa* on protein level induced epileptiform-like discharges in the optic tectum of larval zebrafish brains. Incubation of morphants with antiseizure drugs (sodium valproate, ethosuximide, lamotrigine, topiramate) significantly decreased the number and, in some cases, cumulative duration of epileptiform-like discharges. In this context, sodium valproate seemed to be the least effective. Carbamazepine did not affect the number and duration of epileptiform-like discharges. Altogether, our data indicate that *cacna1aa* loss-of-function zebrafish may be considered a new model of absence epilepsy and may prove useful both for the investigation of *Cacna1a*-mediated epileptogenesis and for in vivo drug screening.

**Electronic supplementary material:**

The online version of this article (10.1007/s12035-019-01860-x) contains supplementary material, which is available to authorized users.

## Introduction

*CACNA1A* encodes the pore-forming α1 subunit of voltage-gated P/Q type Ca^2+^ channels (Ca_v_2.1) [1]. These channels are most abundantly located on presynaptic terminals, especially in Purkinje cells of the cerebellum where they control neurotransmitter release [2–5]. However, high expression of P/Q calcium channels has also been found in the frontal cortex and the CA1 region of the hippocampus [2, 6], the brain structures involved in generation, maintenance and spread of discharges in generalized epilepsy [7]. Mutations in *CACNA1A* have been described in patients suffering from autosomal-dominant diseases: familial hemiplegic migraine type 1, spinocerebellar ataxia type 6, and episodic ataxia type 2 (reviewed by [8]).

Although the type of mutation in the *CACNA1A* gene determines, at least partially, the disease phenotype, mutation carriers still exhibit a diverse range of symptoms, which moderately overlap. It is believed that predominantly nonsense mutations or deletions of the gene determine the clinical manifestations in episodic ataxia type 2 patients [9, 10]. However, missense mutations in *CACNA1A* resulting in loss of P/Q type Ca^2+^ channel activity were described in infantile epilepsy with myoclonus [11]. Apart from recurrent ataxia, incoordination, slurring of speech, vertigo, and/or nystagmus, some patients present absence [12], myoclonic [13, 14], or febrile seizures [13, 15]. Moreover, early-onset epileptic encephalopathy has been described in humans [9, 10, 16]. Additionally, *CACNA1A* mutations have been detected in rodents [17–19] and humans suffering from absence seizures with/without cerebellar ataxia [13, 20, 21].

In the last decade, zebrafish (*Danio rerio*) has emerged as a new, attractive species for modeling human brain disorders. With regard to epilepsy research, the utility of zebrafish to mimic aspects of this human disorder has been demonstrated for Dravet syndrome (i.e., *SCN1A* mutations) [22, 23], pyridoxine-dependent epilepsy (*ALDH7A1* and *PLPBP)* [24, 25]*,* focal seizures *(DEPDC5)* [26], or in *CHD2*-mediated epileptic encephalopathies [27, 28]. More recently, Samarut et al. [29], using CRISPR/Cas9 technology, generated a new *gabra1*^−/−^ mutant zebrafish line in order to unravel the epileptogenic mechanisms underlying *gabra1* deficiency and this study undoubtedly confirmed the potential of zebrafish for elucidating mechanisms underlying the process of epileptogenesis. Additionally, drug screening in zebrafish larvae has also been performed in genetic epilepsy models. Baraban et al. [30] took advantage of the epileptic phenotype of *scn1lab*^−/−^ mutant zebrafish larvae and were able to identify clemizole as a potential new drug candidate by using a large-scale screening program in *scn1lab*^−/−^ mutant zebrafish. Zhang et al. [22] demonstrated that another zebrafish model of Dravet syndrome responded to a drug lead in the same manner as human patients. They showed for the first time the anti-seizure effect of fenfluramine in *scn1lab* knockdown zebrafish larvae. Interestingly, fenfluramine, which showed success in phase III trials for the management of Dravet syndrome, did not exhibit any activity in the equivalent rodent models, highlighting the utility of zebrafish for identifying and/or validating new drug leads. More recently, Sourbron et al. [31] performed a drug-repurposing screen, by assessing the response of *scn1lab*^−/−^ mutant larvae to three different drugs targeting the serotonergic system. In this preliminary study, lisuride (anti-parkinson’s drug, 5-HT_2A_, types 2 and 3 dopamine receptor agonist) emerged as a new drug candidate for Dravet syndrome patients.

With regard to developmental stages, 3-day post-fertilization (dpf) in zebrafish (i.e., day of hatching) corresponds to the time of human birth, while every successive day thereafter corresponds to 3 months of age in humans (i.e., 4, 5, 6, and 7 dpf are the equivalent to 3, 6, 9, and 12 months of age in children, respectively). The zebrafish is also a favorable alternative to rodents in the context of genetic manipulation (described in detail by [32]). The zebrafish genome has been fully sequenced and annotated and exhibits approximately 70% similarity with the human genome [33]. Of note, during the process of evolution, some genes were duplicated in zebrafish [34]. The *cacna1a* gene in zebrafish is duplicated, with 72.01% (*cacna1aa*) and 71.28% (*cacna1ab*) homology with human *CACNA1A* (https://zfin.org/), the former having three splice variants in zebrafish.

Although, there are good models of absence epilepsy in rodents, including the well-established and pharmacologically validated GAERS and WAG/Rij rats, spike-wave discharges in these models start appearing relatively late during development (2–3 months of age, which corresponds to the juvenile stage in humans). This is not consistent with the fact that absence epilepsy in humans typically manifests itself early during development (childhood). Moreover, although WAG/Rij rats exhibit absence seizures, the mutation leading to the epilepsy phenotype has not been identified to date [35]. In case of GAERS rats, it is believed that mutations in *Cacna1h* lead to epilepsy [36]. In this context, the zebrafish model of absence epilepsy may offer another advantage. Thus, in this study, we aimed for the first time to assess whether larval zebrafish may suffer from *cacna1a*-mediated absence seizures. Given that there is a lack of data about *cacn1aa*-related zebrafish phenotypes, we therefore aimed to describe all phenotypic defects in this study. Toward this end, we first assessed the expression of *cacna1aa* in the larval zebrafish brain using in situ hybridization analysis. Next, the combination of two antisense morpholino oligomers (MOs) targeting ATG codons of all splice variants was used to achieve partial knockdown of *cacna1aa*. Using this approach, we assessed whether the partial loss-of-function (LOF) of *cacna1aa* could induce an epileptic-like phenotype in larval zebrafish, both on the behavioral and electroencephalographic (EEG) levels. To further examine the character of *cacna1aa* epileptiform-like discharges, we assessed the activity of four antiseizure drugs (ASDs) effective in the treatment of human absence seizures (i.e., sodium valproate (VPA), ethosuximide (ETX), lamotrigine (LTG), and topiramate (TPR)) and one drug (i.e., carbamazepine (CBZ)) that is contraindicated for this type of seizure.

## Materials and Methods

### Zebrafish Maintenance

Adult zebrafish (*Danio rerio*) stocks of the AB strain (kind gift from Ana Carolina Sulen Tavara, Norwegian University of Life Sciences, Oslo, Norway) were maintained at standard aquaculture conditions (i.e., 28.5 °C, 14/10 h light/dark cycle). Fertilized eggs were collected via natural spawning. Embryos were reared under constant light conditions in embryo medium, i.e., Danieau’s buffer: 1.5-mM Hepes, pH 7.6, 17.4-mM NaCl, 0.21-mM KCl, 0.12-mM MgSO_4_, and 0.18-mM Ca(NO_3_)_2_. All embryos and larvae were kept in an incubator, at 28.5 °C. All experimental protocols and housing conditions were carried out according to the National Institute of Health Guidelines for the Care and Use of Laboratory Animals, the European Community Council Directive of November 2010 for Care and Use of Laboratory Animals (Directive 2010/63/EU), and the ARRIVE guidelines. All experiments were approved by the Norwegian Food Safety Authority experimental animal administration’s supervisory and application system (FOTS-18/106800-1).

### Drugs

The following ASDs were used: CBZ (100 μM), ETX (10 mM), LTG (200 μM), TPR (100 μM), and VPA (100 μM). All drugs, except for VPA (Sanofi Aventis), were purchased from Sigma-Aldrich. The doses of drugs were chosen on the basis of previous literature [22, 37] and preliminary tests. All ASDs were dissolved in DMSO and diluted in embryo medium to achieve a final concentration of DMSO of 0.5% *v*/v. Embryo medium, prepared with DMSO in a final concentration of 0.5% *v*/v, served as a vehicle (Veh).

### Whole-Mount In Situ Hybridization

Whole-mount in situ hybridization analysis for *cacna1aa* was performed as previously described [38] using digoxigenin-labeled riboprobes. Primer sequences for *cacna1aa* sense and antisense probes are plotted in Table 1. Embryos were fixed at 4 or 5 dpf in 4% paraformaldehyde in 1 × PBS. Digoxigenin (DIG) UTP–labeled RNA riboprobes were made from linearized constructs using the mMESSAGE mMACHINE™ SP6 Transcription Kit, mMESSAGE mMACHINE™ T7 Transcription Kit (Thermo Scientific Fisher), and DIG RNA labeling Mix (Roche). Sense and antisense RNA probe-stained 4 dpf and 5 dpf larvae were imaged on a Stemi 508 DOC Zeiss stereomicroscope with mounted axiocam color camera. The experiment was replicated twice, with *n* = 6–8/group.

**Table 1:** Sequences of antisense MOs and primer sequences for *cacna1aa* sense and antisense probes

Morpholino name	Morpholino sequence	Primer sequences for probes
*cacna1aa* MO1	5’-TGTACTCAAATGGAGTGAGAATCAT-3’	f: 5’CCTTGACCTATGATTCTCACTCC3’
*cacna1aa* MO2	5’-TCATCTCCGAACCGAGCCATTCTAT-3’	r: 5’GCACTCCCTGCAGCATCATTGCT3’
Ctrl-MO	5’-CCTCTTACCTCAGTTACAATTTATA-3’	
p53 MO	5’-GCGCCATTGCTTTGCAAGAATTG −3’	

*f*, forward; *r*, reverse

### Microinjection of Antisense MOs

Antisense MOs were designed and synthesized by Gene Tools, LLC (Philomath, OR, USA). The sequences of MO were plotted in Table 1. The targets for partial knockdown were ATG codons of *cacna1aa* transcripts, i.e., *cacna1aa*-201 and *cacna1aa*-202 & *cacna1aa*-203, herein referred to as MO1 and MO2, respectively. A random sequence standard control MO (Ctrl-MO) and p53 MO (4 ng), suppressing p53 mRNA, were used to assess the specificity of the observed phenotype. All MOs, individually or in combination, were injected into the yolk of one- or two-cell stage embryos, in a total volume of 1.5 nl/embryo.

### Western Blot Analysis

The anti-CACNA1A antibody was generated using a synthetic peptide from human CACNA1A amino acids 2050–2150. Using Clustal Omega, alignment of this region showed 89% and 50% homology between human CACNA1A and zebrafish cacna1aa and human CACNA1A and zebrafish cacna1ab, respectively.

Four-day-old Ctrl-MO and *cacna1aa* morphants were collected (25 larvae/sample, *n* = 3–4/group), placed in 100 μl of *RIPA buffer* (Sigma Aldrich), immediately boiled at 95 °C for 10 min and kept at − 80 °C until further analysis. Total protein was separated on a 12% SDS-polyacrylamide gel and transferred electrophoretically to a nitrocellulose membrane. Next, the membrane was blocked for 1 h with 5% skim milk (Sigma-Aldrich, USA) in PBS containing 0.1% Tween-20 and again incubated overnight with rabbit monoclonal anti-CACNA1A antibody (ab181371, 1:2000; Abcam) or rabbit monoclonal anti-ß-actin antibody (ab8226, 1:2000; Abcam) that served as primary antibodies. Goat anti-rabbit (31460, 1:2500; Thermo Fisher) horseradish peroxidase-conjugated secondary antibody was used to detect the primary antibodies, and the resulting signal was measured with the SIGMAFAST™ DAB with Metal Enhancer (SLBP7387V; Sigma Aldrich). Prestained molecular weight protein marker (Presicion Plus Protein™ Dual Color Standars; Bio-Rad) was used to determine the molecular weight of each detected band and to confirm antibody target specificity. Total protein level was normalized relative to ß-actin protein level.

### Morphological Phenotyping

Mortality of eggs and larvae injected with Ctrl-MO and *cacna1aa* MO (individually and in combination) was assessed 4 h post-fertilization (hpf) and 24 hpf. Larvae were visually inspected for severe malformations (e.g., pericardial edema, body axis curvature, hemorrhage) from 1 to 5 dpf. For documentation, *cacna1aa* and Ctrl-MO morphants were photographed at 4 dpf, using a Leica MZ10F stereomicroscope equipped with a DFC310 FX digital camera.

### Touch-Evoked Response

At 4 dpf, the touch-evoked response was evaluated in 48-well plates. Larvae were lightly touched on the tail with the tip of metal tweezers and their response was scored as follows: (1) “absent”—the larva does not move at all after several tactile stimuli given, (2) “decreased”—the larva performs a movement after several stimuli, (3) “normal”—the larva moves immediately after a single stimulus.

### Locomotor Activity

At 4 dpf, *cacn1aa* morphants as well as Ctrl-MO zebrafish larvae were placed in a 48-well plate (one larva per well) filled with 300 μl of embryo medium, and habituated to the automated tracking device (ZebraBox, Viewpoint, Lyon, France) for 10 min in light, followed by 10 min in dark phase. Subsequently, the distance covered in millimeters by each larva was recorded for a total period of 10 min, with the following intervals: (1) 5 min in light phase and (2) 5 min in dark phase. Two independent experiments were done, and the data were pooled together.

### EEG Analysis

In order to detect epileptiform-like discharges in zebrafish larvae, the EEG recordings were conducted according to the method described previously [39]. The EEG recordings were obtained from zebrafish larval optic tectum at 4 dpf. Larvae were immobilized in a thin layer of 2% low-melting-point agarose, and the glass electrode (resistance 1–5 MΩ) filled with artificial cerebrospinal fluid (124 mM NaCl, 2 mM KCl, 2 mM MgSO_4_, 2 Mm CaCl_2_, 1.25 mM KH_2_PO_4_, 26 mM NaHCO_3_, 10 mM glucose) was placed into the optic tectum (MultiClamp 700B amplifier, Digidata 1550 digitizer, Axon instruments, USA). Single recordings for each larva were performed for a period of 20 min. The threshold for detection of epileptiform-like discharges was set at 3× background noise and 150 ms. The data were analyzed with the aid of the Clampfit 10.2 software (Molecular Devices Corporation, USA) and custom-written R script for Windows.

### Effect of ASDs on EEG

Freely swimming 4 dpf Ctrl-MO and *cacna1aa* morphants were incubated with ASDs or Veh, within 2 h before EEG analysis. EEG recordings were conducted as described above. Number of epileptiform-like discharges, cumulative duration of events, and mean duration of events were determined.

### Statistical Analysis

Mortality and touch-evoked response were analyzed using two-sided Fisher’s exact test. Locomotor activity and EEG data were analyzed by one-way analysis of variance (ANOVA), followed by Tukey’s *post-hoc* test. *P* values less than 0.05 were considered statistically significant. For a purpose of statistical analysis, GraphPad Prism 7.05 version (San Diego, CA, USA) was used. For figures generation, GraphPad Prism 7.05 or ImageJ (https://imagej.nih.gov/ij/) were used.

## Results

### Expression Pattern of *cacna1aa* in the Brain

**Fig. 1:**
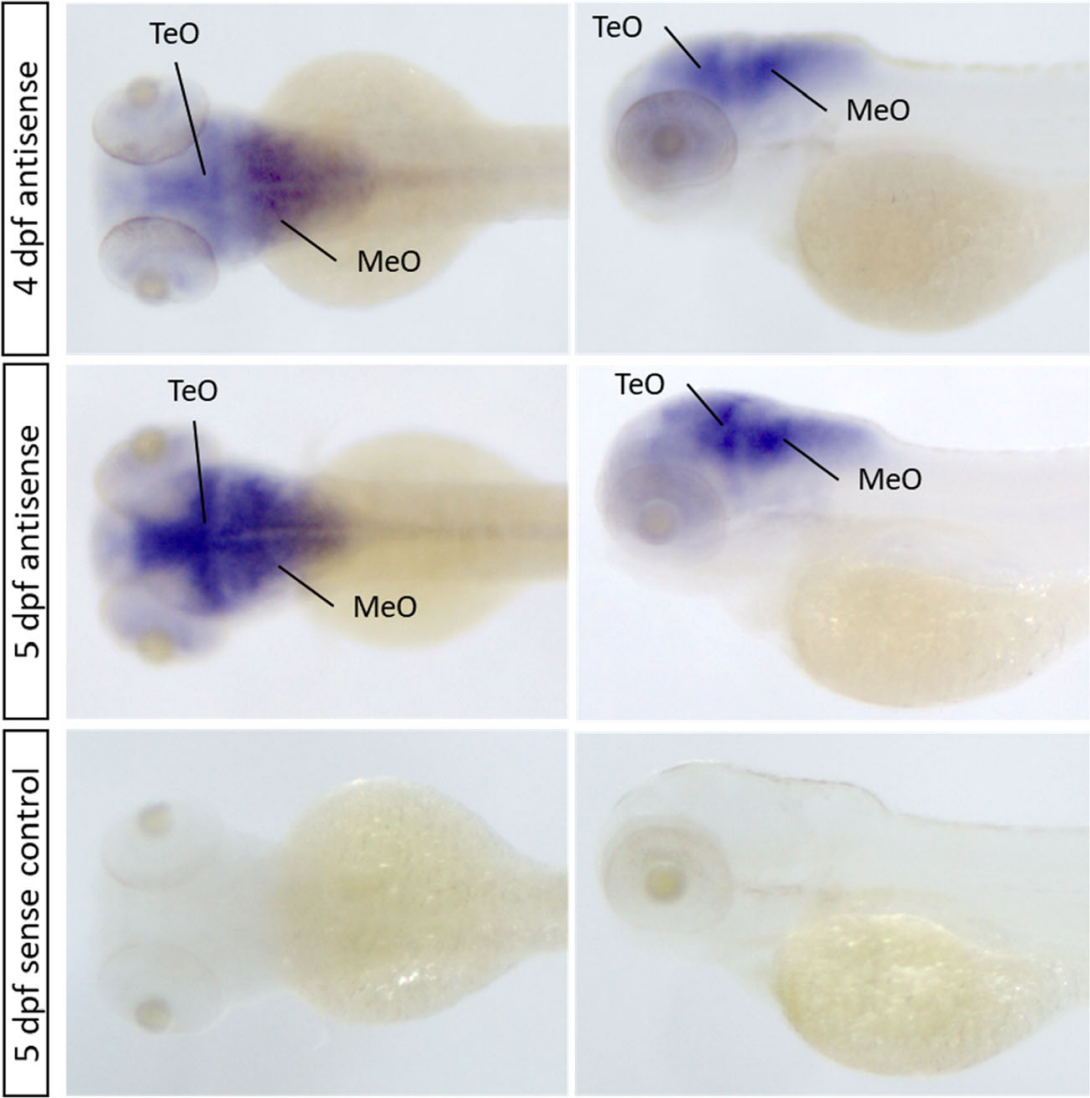
Representative wild type larva in situ hybridization with *cacna1aa* antisense and sense probes. Dpf, days post-fertilization; MeO, medulla oblongata; TeO, optic tectum

Since the expression of *cacna1aa* was previously described by Thisse and Thisse [38] during early stages of zebrafish development, we focused on analyzing cacna1aa mRNA expression at the stages when behavioral and EEG experiments were performed. Both dorsal and lateral views of the head, in whole-mount in situ hybridization, for wild type 4 and 5 dpf zebrafish revealed prominent *cacna1aa* mRNA expression in the midbrain and hindbrain, but low expression in the forebrain and retina (Fig. 1). High expression of *cacna1aa* mRNA at 4 dpf larvae was detected in the optic tectum and even higher expression in the medulla oblongata. The staining was even more pronounced at 5 dpf. No detectable expression was observed in 5 dpf sense control larvae (Fig. 1).

### Mortality Rate and Touch Response of *cacna1aa* Morphants

Microinjected Ctrl-MO affected neither mortality rate of embryos and larvae, assessed at 4 hpf and 24 hpf, nor larval touch response as determined at 4 dpf (Tables 2 and 3).

**Table 2:** Mortality of zebrafish *cacna1aa* knockdown larvae

Treatment	Dose	Death after 4 hpf (%)	Death after 24 hpf (%)
		(n/N)	(n/N)
Wild type	Uninjected	5.29	7.20
		(25/472)	(34/472)
Ctrl-MO	5 ng	2.63	7.89
		(4/152)	(12/152)
*cacna1aa* MO1	7.5 ng	4.33	11.41
		(11/254)	(29/254)
	9 ng	9.39*	43.19*
		(20/213)	(92/213)
*cacna1aa* MO2	9 ng	4.69	11.18%
		(21/447)	(50/447)
	12 ng	10.56*	12.19
		(13/123)	(15/123)
*cacna1aa* MO1 + MO2	2.5 ng + 2.5 ng	4.14	8.03
		(16/386)	(31/386)
	4.5 ng + 4.5 ng	4.69	60.40*
		(7/149)	(90/149)

*n*, number of dead larvae; *N*, total number of larvae; *Hpf*, hours post-fertilization

Statistical analysis was performed using two-sided Fisher’s exact test

*P < 0.05 vs respective Ctrl-MO group

**Table 3:** Touch-evoked response of zebrafish *cacna1aa* knockdown larvae

Treatment	Dose	Normal (%)	Decreased (%)	Absent (%)
		(n/N)	(n/N)	(n/N)
Wild type	Uninjected	97.22	2.78	0
		(35/36)	(1/36)	(0/36)
Ctrl-MO	5 ng	98.07	1.92	0
		(51/52)	(1/52)	(0/52)
*cacna1aa* MO1	7.5 ng	73.37*	18.51*	11.11*
		(38/54)	(10/54)	(6/54)
	9 ng	0*	16.66*	83.33*
		(0/24)	(4/24)	(20/24)
*cacna1aa* MO2	9 ng	52*	13.79*	36.20*
		(29/58)	(8/58)	(21/58)
	12 ng	0*	5.71	94.28*
		(0/35)	(2/35)	(33/35)
*cacna1aa* MO1 + MO2	2.5 ng + 2.5 ng	96.82	3.17	0
		(61/63)	(2/63)	(0/63)
	4.5 ng + 4.5 ng	50*	20.83*	29.16*
		(12/24)	(5/24)	(7/24)

*n*, number of animals with a defined (i.e., absent, decreased, or normal) response; *N*, total number of animals

Statistical analysis was performed using two-sided Fisher’s exact test

*P < 0.05 vs respective Ctrl-MO group

Administration of *cacna1aa* MO1 at 7.5 ng also did not affect mortality rate of embryos and larvae as well, but a higher dose of 9 ng significantly enhanced mortality (*P* < 0.05). Similarly, a second MO, *cacna1aa* MO2, did not influence mortality at both tested time points at the same dose of 9 ng, but slightly increased mortality of embryos and larvae at 12 ng by 4 hpf (*P* < 0.05). Simultaneous administration of *cacna1aa* MO1 + MO2 at combined doses of 2.5 ng + 2.5 ng resulted in no increase in mortality. However, a significant increase in mortality to 60% was evoked by simultaneous administration of *cacna1aa* MO1 + MO2 at combined doses of 4.5 ng + 4.5 ng after 24 hpf (Table 2).

With regard to larval touch response, administration of *cacna1aa* MO1 at tested doses of 7.5 ng and 9 ng or of *cacna1aa* MO2 at doses of 9 ng and 12 ng hampered larval touch response significantly. Simultaneous administration of *cacna1aa* MO1 + MO2 at doses of 2.5 ng + 2.5 ng did not influence touch response, whereas combined doses of 4.5 ng + 4.5 ng resulted in a moderate decrease in the touch-evoked response (Table 3).

**Fig. 2:**
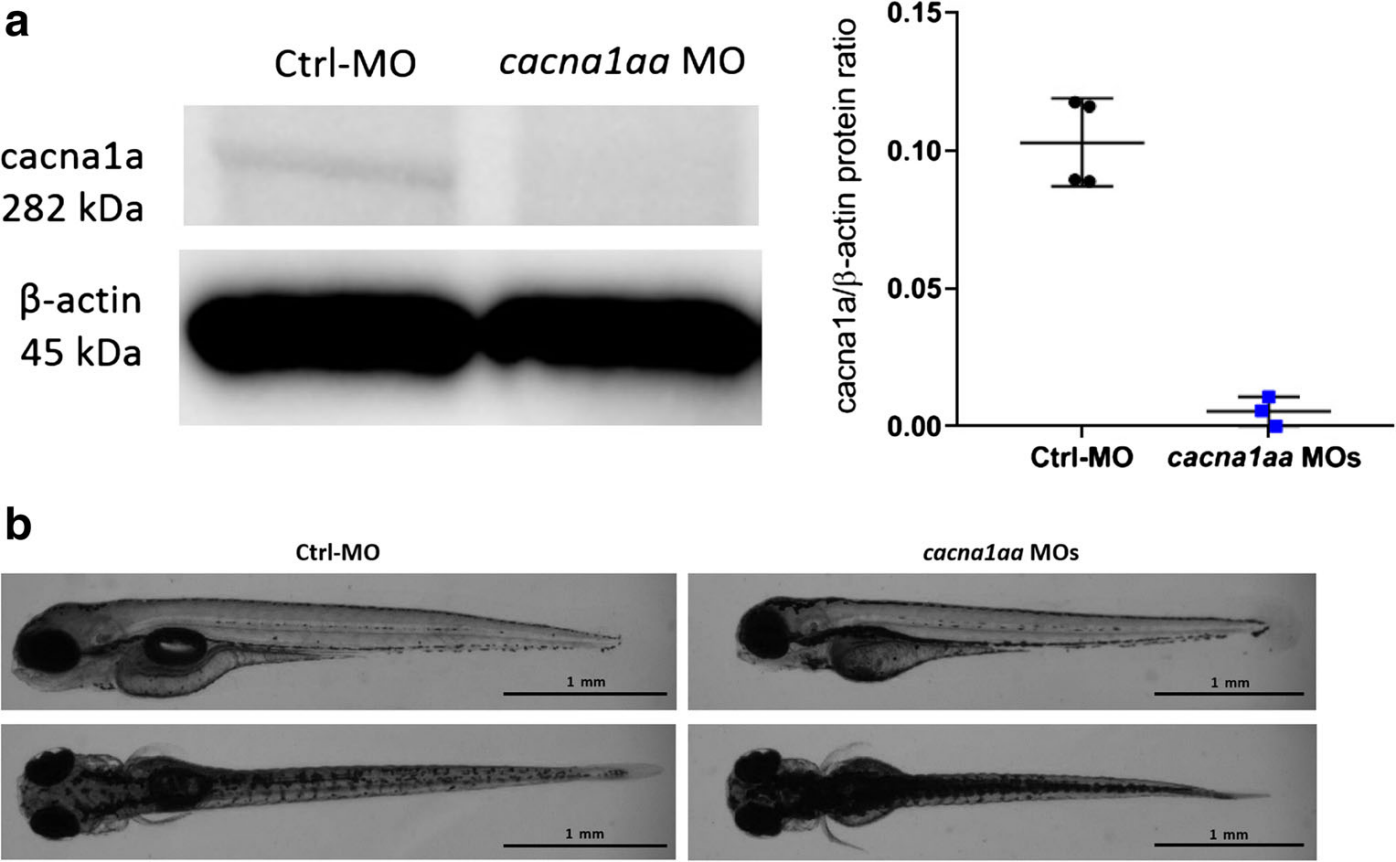
**a** Representative western blot of 4 dpf *cacna1aa* and Ctrl-MO larvae (left panel) and quantification of all samples (right panel). **b** Dorsal and side views of representative 4 dpf Ctrl-MO and *cacna1aa* MOs larvae

Based on these results, the simultaneous administration of *cacna1aa* MO1 + MO2 at doses of 2.5 ng + 2.5 ng was chosen for further experiments. The western blot analysis revealed that these doses reduced cacn1aa protein levels to 10% relative to the control (Fig. 2a and Supp. Fig 1).

### Morphological and Behavioral Assessment of *cacna1aa* Morphants

Visual inspection of *cacna1aa* morphants (9 or 12 ng, single dose) that survived until 4 dpf revealed profound morphological malformations (curved body axis, small heads, tiny eyes, pericardial edema, yolk sac malformations) (data not shown). On the other hand, visual inspection of *cacna1aa* morphants (doses 2.5 ng + 2.5 ng) revealed that at 4 and 5 dpf, larvae were slightly hyperpigmented in comparison with Ctrl-MO counterparts (Fig. 2b). In addition, at 4 dpf, the majority of *cacna1aa* morphant larvae (i.e., 84.4% (38/45) vs. 4.2% (1/24) of Ctrl-MO injected larvae) did not inflate their swim bladder (see Fig. 2b). Measurements taken at 4 dpf revealed that *cacna1aa* morphants had a shorter body length (*P* < 0.05) compared with Ctrl-MO larvae (3.51 mm ± 0.15, *n* = 11 vs 3.73 mm ± 0.12, *n* = 9, respectively) (data not shown). There were no observable signs of necrosis, hemorrhage, pericardial edema, or axis truncation. The simultaneous administration of *cacna1aa* MOs and p53 MO (4 ng) indicated that the observed morphological changes were likely specific to *cacna1aa* partial knockdown and not due to off-target effects of the MO itself (see Supp. Fig 2).

**Fig. 3:**
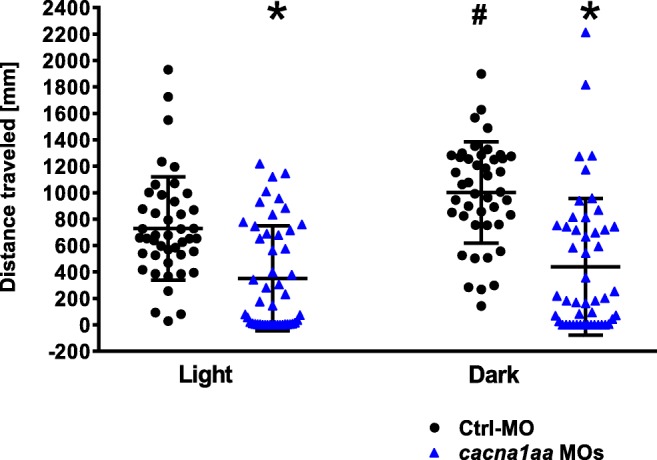
Locomotor activity of 4 dpf *cacna1aa* and Ctrl-MO larvae. Larvae were habituated to the apparatus 20 min (10 min light, 10 min in dark) before experiment, and total locomotor activity was tracked within 10 min. The data were pooled from 2 independent experiments. The results were analyzed using one-way ANOVA, followed by Tukey’s *post-hoc* test. Dots represent individual measurements, the central horizontal mark is the mean, and error bars represent standard deviation (SD) (*n* = 44–48/group). ^*^P < 0.05 vs Ctrl-MO group in relevant phase, ^#^*P* < 0.05 vs Ctrl-MO group in light phase. Dpf, days post-fertilization

To investigate the impact of *partial cacna1aa LOF* on behavior, larval locomotor activity of *cacna1aa* morphants and Ctrl-MO was evaluated at 4 dpf. One-way ANOVA revealed a statistically significant differences between groups of animals (F(3,180) = 21.84, *P* < 0.05, *n* = 44–48/group; Fig. 3). We observed that the switch from light to dark phase increased activity of Ctrl-MO (*P* < 0.05). However, reduced locomotor activity was observed in *cacna1aa* morphants when compared with Ctrl-MO siblings in both analyzed phases (Light: *P <* 0.05, Dark: *P* < 0.05; Fig. 3). Rapid switching from light to dark phase did not increase locomotor activity of morphants (*P* > 0.05).

### Assessment of EEG Discharges in *cacna1aa* Morphants and Ctrl-MO Larvae and Effect of ASDs

**Fig. 4:**
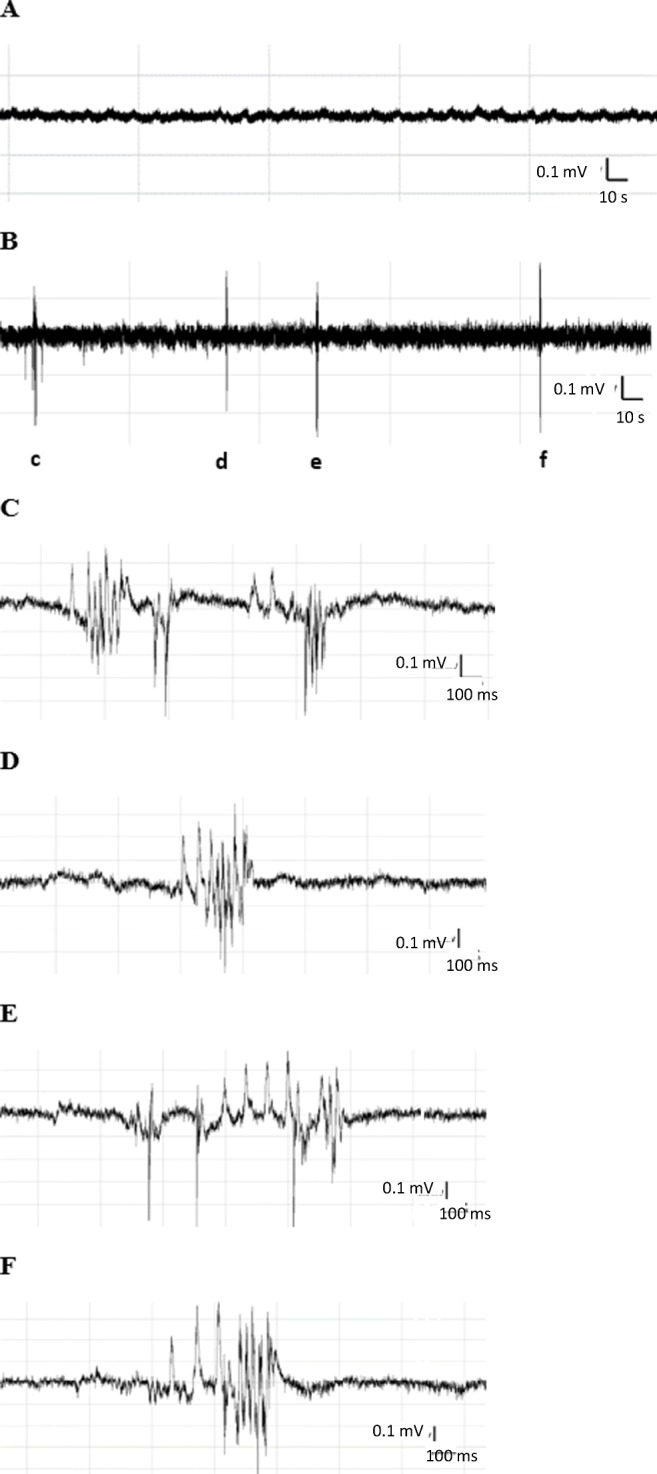
Representative electroencephalographic recording illustrating the epileptiform-like discharges recorded in zebrafish Ctrl-MO larvae and *cacna1aa* morphants (2.5 ng + 2.5 ng). The EEG recordings were obtained from zebrafish larval optic tectum at 4 dpf. **a** Five-minute-long fragment of representative recording from Ctrl-MO larvae. **b** Five-minute lasting continuous recording from *cacna1aa* morphant demonstrating the background and ictal activity. Small letters (c–f) correspond to respective ictal events depicted on traces **b**–**e**. **c**–**f** Epileptiform-like discharges, high-voltage spikes, spike-wave complexes, and polyspike-wave discharges

**Fig. 5:**
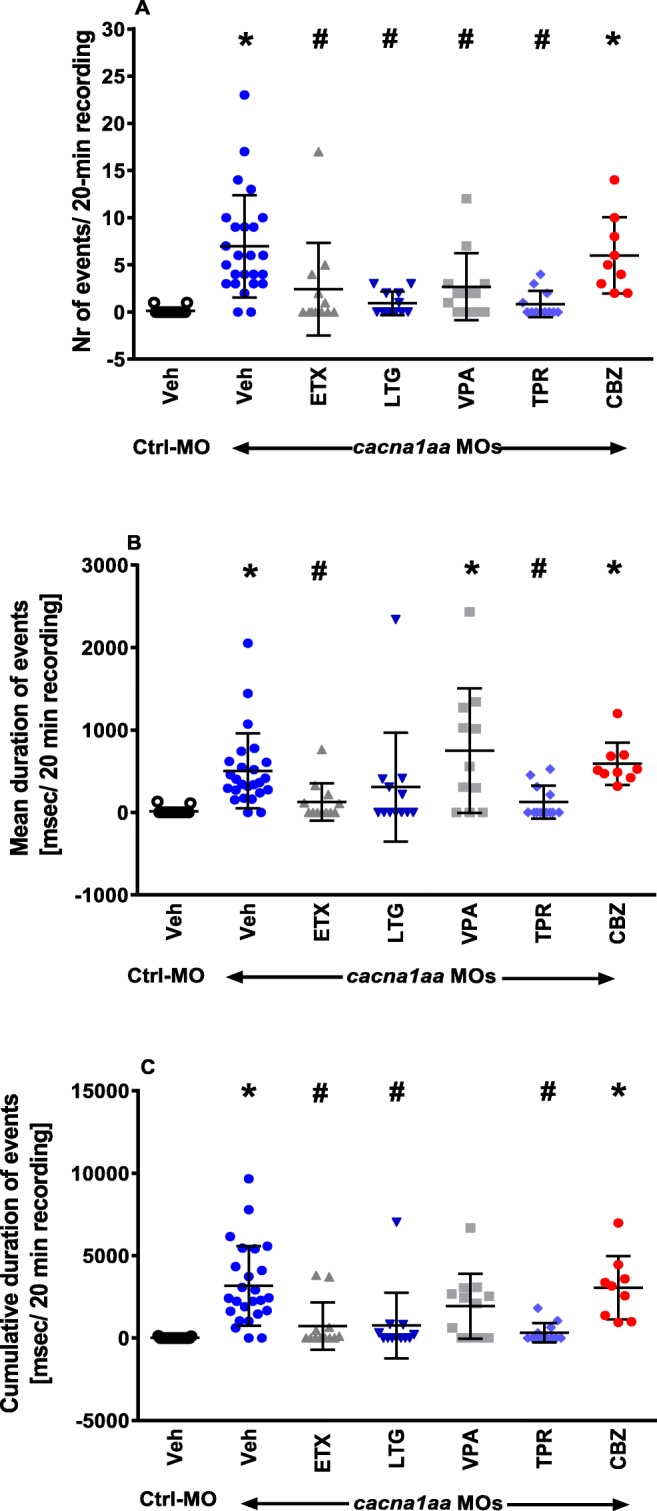
Effect of ASDs on epileptiform-like discharges recorded from the optic tectum of 4 dpf *cacna1aa* and Ctrl-MO morphants. Larvae were incubated (2 h) with different ASDs. Results are presented as **a** number of events, **b** mean duration of events (msec), and **c** cumulative duration of events (msec) during 20 min of recording. Statistical analysis was performed using one-way ANOVA with Tukey’s *post-hoc* test. Dots represent individual measurements, the central horizontal mark is the mean, and error bars represent SD (*n* = 9–25). Symbols represent following comparisons: *P < 0.05 vs Ctrl-MO group incubated with Veh, ^#^*P* < 0.05 vs *cacna1aa* MOs group incubated with Veh. CBZ, carbamazepine (100 μM); ETX, ethosuximide (10 mM), LTG, lamotrigine (200 μM), TPR, topiramate (100 μM), VPA, sodium valproate (100 μM)

In 2 out of 19 (10.5%) control morphants, single short-duration epileptiform-like events were observed (Fig. 4a). Spontaneous epileptiform-like events in the form of abrupt high-voltage spikes, spike-wave complexes, and polyspike-wave discharges (Fig. 4b–f) occurred in 24 out of 26 (92%) *cacna1aa* morphants. In seizure-positive *cacna1aa* morphants, a mean frequency of events was 7 events/20-min recording, compared with 0.5 events/20-min recording in Ctrl-MO counterparts (Fig. 5a). In *cacna1aa* morphants, the mean and cumulative duration of EEG discharges was 503 and 3164 ms/20 min, respectively (Fig. 5b–c). One-way ANOVA revealed statistically significant differences between the tested groups in the number of epileptiform-like discharges (F(6.94) = 9.16, *P* < 0.05; *n* = 9–25/group; Fig. 5a), mean duration of events (F(6.94) = 5.64, *P* < 0.05; *n* = 9–25/group; Fig. 5b), and cumulative duration of events (F(6.94) = 9.17, *P* < 0.05; *n* = 9–25/group; Fig. 5c).

Tukey’s *post-hoc* test revealed that 2-h incubation of *cacna1aa* morphants with ASDs indicated for the treatment of absence seizure in humans prior to EEG assessment decreased the number of epileptiform-like discharges substantially, compared with *cacna1aa* morphants incubated with Veh (Fig. 5a). Herein, VPA, ETX, LTG, and TPR were equally potent (*P* < 0.05). However, analysis of mean and cumulative duration of events revealed that ETX and TPR were the most effective in decreasing the duration of these parameters, when compared with Veh-treated *cacna1aa* morphants (*P* < 0.05; Fig. 5 b and c). LTG tended to decrease mean duration of events (*P* > 0.05; Fig. 5b) and decrease the cumulative duration of events (*P* < 0.05; Fig. 5c). Differently, VPA tended to increase the mean duration of events (*P* > 0.05; Fig. 5b) while decreasing cumulative duration of events, but results did not reach statistical significance (*P* > 0.05; Fig. 5c). CBZ, which is contraindicated in human patients with absence seizures, did not decrease any of the parameters when compared with *cacna1aa* morphants incubated with Veh (*P* > 0.05; Fig. 5a–c). Incubation of Ctrl-MO larvae with all ASDs did not induce any changes in their EEG activity, compared with Ctrl-MO larvae incubated with Veh only (*P* > 0.05; *n* = 7–25; Supp. Fig 3 A–C).

## Discussion

Our study revealed that partial *cacna1aa* LOF in larval zebrafish results in profound behavioral impairment and irregular repeated epileptiform-like discharges as measured by EEG. *cacna1aa* LOF in larval zebrafish leads to mortality, even when individual splice variants are targeted. The cause of mortality may be attributable to defects in the brain, peripheral effects, or both. Peripheral effects of *cacna1aa* LOF (2.5 ng + 2.5 ng) were evidenced by changes in morphology of morphants, i.e., slight hyperpigmentation, lack of swim bladder, and shorter body length—phenotypes also observed in other zebrafish models of epilepsy [22, 40]. Notably, cacna1aa mRNA in zebrafish embryos is maternally contributed [41, 42], possibly accounting for the higher mortality rate at 24 h than 4 h after injection. Saito et al. [43] revealed that knockdown of *Cacna1a* to 28% of baseline Ca_v_2.1 induced severe ataxia, while reduction to 14% dramatically shortened the lifespan of mice. Similarly, conditional ablation of Ca_v_2.1 channels leading to *Cacna1a* gene LOF in mice resulted in ataxia and dystonia starting at around postnatal days 10–12 and death at postnatal days 21–22 [44]. In our experimental setting, the cause of mortality is partially related to morphology abnormalities. However, lethal/deleterious effects on brain function cannot be ruled out, especially since our study was based on analyzing behavioral and EEG changes at lower, non-lethal doses of MOs.

The behavioral impairments observed in *cacna1a* morphants indicate defects of neuronal origin. The decreased/absent reflex to the touch stimulus may depend on disruption of the spinal reflex mechanism since the touch-evoked locomotor activity response allows assessment of muscle performance in zebrafish. Touch stimuli, e.g., touch of the zebrafish tail tip with a needle, results in muscle contraction accompanied with burst swim [45]. P/Q calcium channels in mammals are abundantly expressed in neuromuscular junctions where they control presynaptic acetylcholine release [46] and are considered to mediate fast neuromuscular neurotransmission also in zebrafish [47, 48]. To the best of our knowledge, *cacna1aa* expression in muscles and the spinal cord has not been investigated, but *cacna1ab*, the paralog to *cacna1aa*, was proposed as a mediator of locomotor behavior and touch-evoked motor response as indicated in *fakir* mutants [49, 50]. The high expression of *cacna1ab* was found in sensory neurons, but MO knockdown of *cacna1ab* disturbed touch-evoked activation of motor neurons, the third neuron of the reflex arc [50].

Although *cacna1aa* morphants did not inflate their swim bladder at 4 dpf, other reports have indicated that this defect does not necessarily interfere significantly with total distance traveled, but only affects slow movements associated with maintaining balance [22, 51]. Thus, the lack of an inflated swim bladder can likely be ruled out as a cause for reduced locomotor activity.

Reduced locomotor activity in morphants was similar in both light and dark phases. It has been repeatedly demonstrated that abrupt switching from light to dark induces a rapid increase in locomotor activity of zebrafish larvae, which decreases to baseline after a few (5–10) minutes [51, 52]. A similar uniform reduction in motility may indicate the reduction of skeletal muscle reactivity or muscle relaxation due to decreased activity of P/Q calcium channels [47, 48]. On the other hand, Samarut et al. [29] observed reduced locomotor activity in *gabra1*^−/−^ zebrafish mutants. Although rapid switching of light to dark induced a very quick and transient increase in their activity, this was followed by profound hypolocomotion [29].

In this study, we observed that spontaneous epileptiform-like events in the form of abrupt high-voltage spikes, spike-wave complexes, and polyspike-wave discharges occurred in 92% of *cacna1aa* morphants. Previous studies indicated that chemicals (e.g., pentylentetrazole or allylglycine) [37, 39, 53] as well as mutations in different genes (e.g., *scn1lab*, *aldh7a1*) [22, 24] increase locomotor activity in zebrafish larvae reminiscent of tonic-clonic-like seizures. Indeed, tonic-clonic-like seizures have been described both in genetic and pharmacological models of epilepsy in zebrafish [22, 54]. The number and frequency of EEG discharges correlated with behavioral outcome, i.e., a dramatic increase in locomotor activity with rapid “whirlpool-like” circling followed by loss of posture. *cacna1aa* morphants did not display this kind of behavior (thus ruling out tonic-clonic-like seizures), and the frequency of epileptiform-like discharges was lower than in all above-mentioned models. Notably, our data are more in line with the phenotype reported for zebrafish *tsc*^−/−^ mutants (model of tuberous sclerosis complex) [51], with regard to seizure duration and frequency. In this study however, the authors did not draw any final conclusion as to what type of seizures were observed in *tsc*^−/−^ mutants.

In humans and rodents, absence seizures occur as bilateral, synchronous slow-wave discharges (typically 3 Hz or 5–7 Hz, respectively) [12, 17, 19–21, 55]. Although we could not clearly distinguish this pattern of discharges in *cacna1aa* morphants, we cannot rule out that species differences may determine the EEG pattern of absence seizures in zebrafish.

Our study revealed that all four drugs recommended in the management of absence seizures significantly diminished the number of epileptiform-like events in 4-dpf *cacna1aa* morphants and were equally potent. Noteworthy, VPA did not affect mean and cumulative duration of events, with the tendency to even increase the latter. Interestingly, the International League Against Epilepsy (ILAE) guidelines recommend VPA as the drug of choice for the treatment of absence epilepsy (class I) in children [56]. On the other hand, we observed that TPR was equally potent when compared with ETX, a class I–indicated drug for absence seizures. Although, ILAE guidelines do not include recommendations for TPR, it is commonly used to treat absence seizures in humans as a second-choice therapy [57, 58]. LTG belongs to class III (i.e., possibly effective) ASDs for absence seizures in patients. In our model, LTG significantly reduced the cumulative duration of EEG discharges and exhibited a tendency to decrease mean duration of events. In rodents with *Cacna1a* LOF, both ETX and VPA efficacy were observed [17, 18, 59] with less consistent data for other drugs, i.e., LTG and TPR [60, 61]. ETX also decreased the incidence of EEG discharges in WAG/Rij rats [62–64]. Nevertheless, taking our data into consideration, it is possible that differences in ASD modes of action might account for differences in our observations. ETX is believed to selectively inhibit T-type calcium channels, while VPA has broader spectrum activity and apart from calcium channel inhibition, also exerts its effect through sodium channel inhibition and activation of GABA-ergic neurotransmission. LTG is a broad-spectrum blocker of calcium and sodium channels, increasing GABA levels, while TPR additionally increases the affinity of GABA to GABA_A_ receptors (for review see [65]). CBZ, also a sodium channel blocker, did not affect the number of EEG discharges in *cacna1aa* morphants. It was reported that CBZ might exaggerate absence seizures in *Cacna1a* mutation carriers, both in humans and rodents [66, 67]. Similarly, CBZ exaggerated the incidence of EEG discharges in WAG/Rij rats [63]. On the other hand, phenytoin, another sodium channel blocker, did not affect seizure incidence in *Cacna1a* models of absence seizures [17, 18, 59]. Although, CBZ did not increase the number of EEG discharges in *cacna1aa* morphants, it cannot be excluded that the time of incubation (2 h) was not long enough to enhance EEG discharges.

In summary, our study describes for the first time, the phenotypic profile of reduced *cacna1aa* function in larval zebrafish, which causes significant locomotor impairment and even lethality. The described behavioral phenotype is accompanied by irregular, repeated epileptiform-like discharges as recorded by EEG. In addition, the pharmacological profiling data further support the validation of this new model for the study of absence seizures. Given that absence epilepsy is most common in children, the use of a developmental model such as the zebrafish allows for the study of epileptogenesis mechanisms (e.g., observed differences in cortical interneuron populations) [68] in the brain at relevant life stages when absence seizures are most likely to occur. Our analysis of the *cacna1aa* LOF in zebrafish was performed at 4 dpf, which is equivalent to 3 months post-birth in humans. This larval zebrafish model is also amenable to large-scale drug screening, opening up the possibility for the discovery of new therapeutic compounds to treat the 30% of absence epilepsy patients that are drug resistant. Furthermore, although absence epilepsy is categorized as a relatively benign form of epilepsy compared with other genetic generalized epilepsy syndromes, it is still often accompanied by comorbidities that can persist even after seizure freedom is achieved. Moreover, drugs used to treat absence epilepsy, such as ETX and VPA, are often not well tolerated or are ineffective. Thus, this zebrafish model can also be used to test for potential disease-modifying activity of drugs with regard to effective treatment of comorbidities (e.g., learning and memory deficits, attention deficit hyperactivity disorder, and anxiety) associated with absence epilepsy.

## Electronic supplementary material

ESM 1 (DOCX 838 kb)
