# Supplementary material for: Phenotypic Characterization of Larval Zebrafish (Danio rerio) with Partial Knockdown of the cacna1a Gene
Source: Mol Neurobiol. 2019 Dec 26;57(4):1904–16. doi: 10.1007/s12035-019-01860-x (PMC7118054; doi:10.1007/s12035-019-01860-x)
Supplement: Supplementary file 1 — (DOCX 838 kb) [file 12035_2019_1860_MOESM1_ESM.docx]

**Supplementary figures**

**Fig. 1.** Western blot of 4 dpf *cacna1aa* MOs and Ctrl-MO larvae. KD i.e. *cacna1aa*-injected morphants.
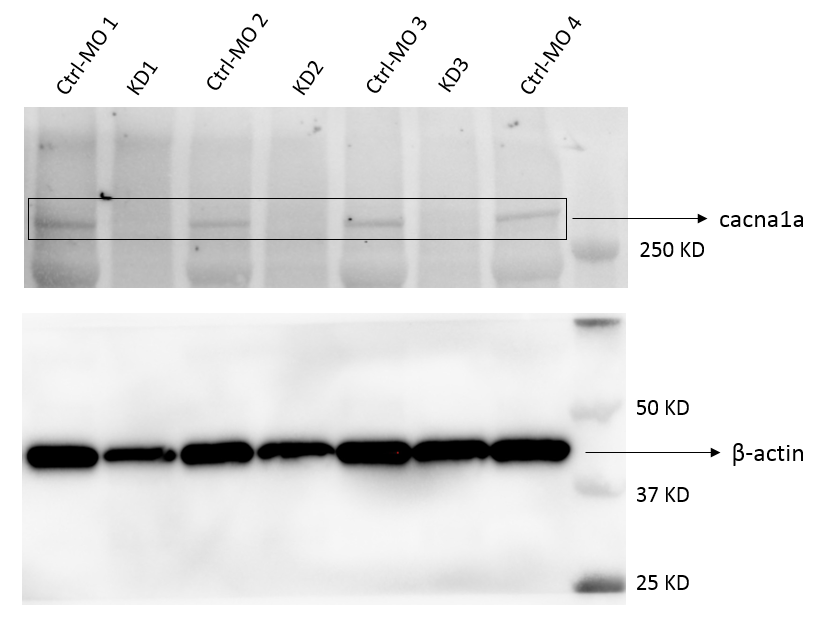


**Fig. 2.** Dorsal and side views of representative 4 dpf Ctrl-MO and *cacna1aa* MOs larvae, co-injected with p53 MO (4 ng).


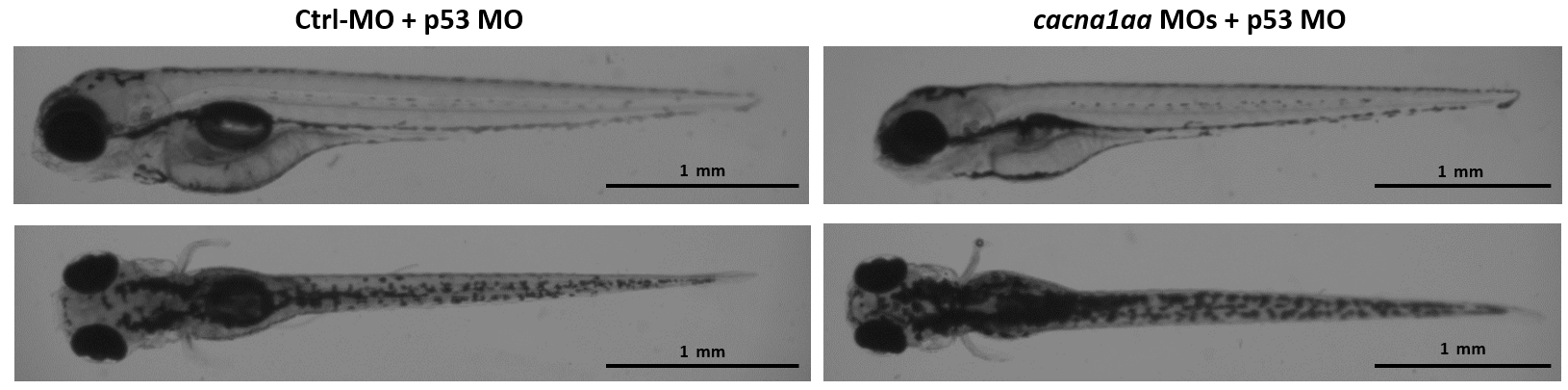


**Fig. 3.** Effect of ASDs on epileptiform-like discharges recorded from the optic tectum of Ctrl-MO morphants. Larvae were incubated (2 h) with different ASDs. Results are presented as (A) number of events, (B) mean duration of event [msec] and (C) cumulative duration of events [msec] during 20 min of recording. Statistical analysis was performed using **one-way** ANOVA with **Tukey’s** *post-hoc* test. **Dots represent individual measurements, the central horizontal mark is the mean and error bars represent SD (n=7-25/group).** CBZ- carbamazepine (100 µM); ETX- ethosuximide (10 mM), LTG- lamotrigine (200 µM), TPR- topiramate (100 µM), VPA- sodium valproate (100 µM).
